# Infrared Efficiency and Ultraviolet Management of Red-Pigmented Polymethylmethacrylate Photoselective Greenhouse Films

**DOI:** 10.3390/polym14030531

**Published:** 2022-01-28

**Authors:** Norah Alwadai, Samah El-Bashir

**Affiliations:** 1 Department of Physics, College of Sciences, Princess Nourah bint Abdulrahman University, P.O. Box 84428, Riyadh 11671, Saudi Arabia; nmAlWadai@pnu.edu.sa; 2 Department of Physics, Faculty of Science, Benha University, Benha 13518, Egypt

**Keywords:** PMMA, perylene, CIE 1931, UV-blocking effect, UV-open effect, thermic effect

## Abstract

Red-pigmented photoselective polymethylmethacrylate (PMMA) films were prepared by casting from polymer/chloroform solution. The films were doped with efficient red fluorescent perylene dyes specialized for plastic coloration, namely KREMER 94720 and KREMER 94739, which have excellent weathering stability and a high fluorescence quantum yield. The effect of the doping concentration was studied using the atomic force microscope (AFM), optical transmission, color measurement, time-resolved fluorescence, and Fourier transform infrared spectroscopy (FTIR). The obtained results suggested the potential usefulness for photoselective greenhouse cladding applications as the lowest doping concentration (10^−5^ wt%) displaying the UV-open effect, whereas the best UV-blocking and thermic effects were obtained for the highest doping concentration (10^−1^ wt).

## 1. Introduction

**Figure 1:**
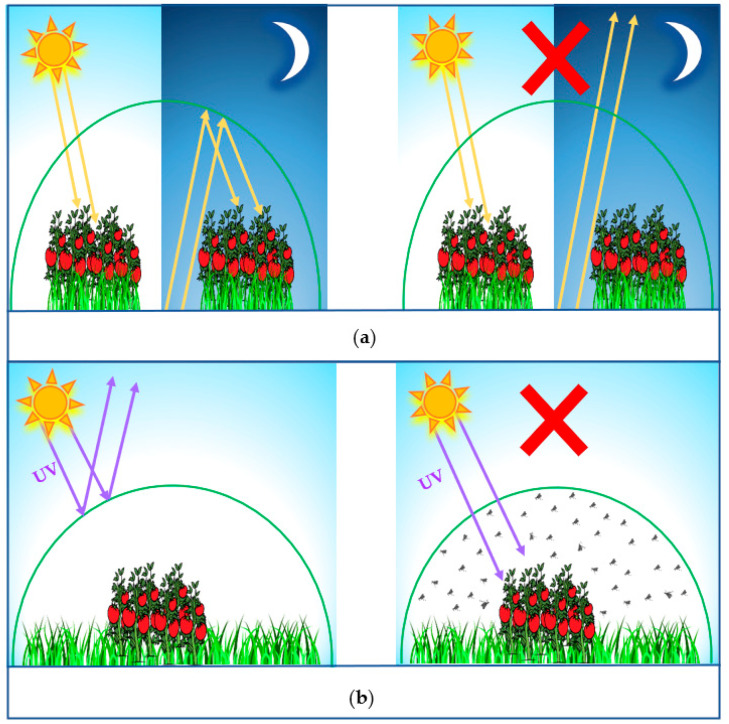
The new features of the suggested photoselective PMMA greenhouse films doped with perylene dyes KREMER 94720 and KREMER 94739. (**a**) Thermic effect. (**b**) UV-blocking effect.

For the past decades, plastic sheets have been used to protect the plants inside greenhouses against severe weather conditions, such as frosty cold and scorching sun. These sheets had owed their success in greenhouse construction to their physical properties, such as high-light transmittance, superior strength, light weight, low cost, heat insulation, as well as weathering resistance [1,2]. There are different types of greenhouse plastic materials such as polyethylene (PE), polyvinylchloride (PVC), polymethylmethacrylate (PMMA), and polycarbonate (PC) [3,4,5]. PMMA, which is commercially known as acrylic, has been widely used for greenhouse glazing due to its unique physical characteristics; it has very high transmittance and scratch resistance compared to PC [6]. Recently, a range of special photoselective PMMA greenhouse films has been proposed, including the pigmentation of greenhouse claddings to modify the sunlight spectrum entering the greenhouse in order to match the action spectrum of chlorophylls required for photosynthesis active radiation (PAR) [7,8,9,10,11,12]. The idea of these photoselective films depends on using integrated luminescent solar concentrator (LSC) technologies for greenhouse applications [13]. LSCs are distinctive non-imaging optical devices that can be used to concentrate sunlight onto photovoltaic (PV) cells. A typical LSC design consists of a transparent plate (e.g., PMMA) doped with luminescent materials such as organic dyes with PV cells optically matched to the plate edges [14,15]. LSCs were firstly proposed in the late 1970s [16,17,18], and then studied intensely through the early 1980s until the thermodynamic limitations of luminescent organic dyes hindered the further development of LSC efficiency [19,20,21]. In the early 1990s, the development of perylene luminescent dyes has renewed the research interest in LSCs [22,23,24] due to their fluorescence efficiency and long-term photostability. Recently, perylene dyes have been used successfully for doping LSCs for a wide range of promising applications, such as solar dryers [5], smart windows [25], greenhouses [26], algae production [27,28], chemical reactors [29], and solar power generation for building-integrated photovoltaics [30,31]. Regarding LSC greenhouse applications, the luminescent perylene dye molecules absorb a considerable fraction (about 40%) of the PAR spectrum in the range of 400–700 nm, which is not absorbed by chlorophylls and fluorescence in the deep red range is necessary for the photosynthesis process [12]. The main advantage of using LSC as greenhouse claddings is the enhancement in the effective transmission of PAR light due to the efficient collection of both direct and diffuse sunlight as well [4]. The present work introduced new features for different compositions of photoselective greenhouse films based on PMMA doped with perylene dyes KREMER 94720 and KREMER 94739. These dyes have unique physical properties such as excellent photostability, intense absorbance in the green–yellow band, and fluorescence efficiency in polymer matrices [5,26]. Besides the photoselective effect, PMMA greenhouse films introduced new spectral features such as the UV-open, the UV-block, and thermic effects, which can be controlled by the dye concentration according to the needs of each crop, as illustrated by the sketches shown in Figure 1. In the case of the UV-open effect, the film allows the highest near-UV transmission (>95%) in the wavelength range of 300–400 nm and provides the same sunlight illumination conditions as outside the greenhouse. Moreover, UV-open films are important for increasing the number of active substances (e.g., vitamin D, phenolics, etc.) and also for the pollination of plants inside the greenhouse because the bumblebee needs UV radiation to navigate [32]. On the other hand, the effect of UV-blocking helps to control pests inside the greenhouse due to the elimination of the wavelengths of up to 380 nm, causing insects to become disoriented [33,34,35]. This property is also important for effective disease control and for the reduction of the blackening of crops due to the accumulation of dark pigments [35,36]. Regarding the thermic effect, infrared radiation is trapped inside the greenhouse and cannot escape during the night to protect the plants from sudden temperature drops, especially for greenhouses built in cold climates [37,38]. 

## 2. Experimental Techniques

### 2.1. Preparation of Photoselective PMMA Greenhouse Films

Photoselective PMMA greenhouse films of 50 ± 10 µm were prepared as described in our previous work by casting from polymer solutions prepared by dissolving PMMA grains (purity 99.9%, SABIC, Riyadh, Saudi Arabia) and red fluorescent perylene dyes separately in chloroform [26]. Two kinds of perylene dyes were used, namely KREMER 94720 and KREMER 94739 (purity 99.9%, Kremer Pigmente GmbH & Co., Aichstetten, Germany), with five concentrations ranging from 10^−5^ to 10^−1^ wt%, giving different transparent colors ranging from light to deep red [26]. The samples were cut into rectangular pieces with about a 4 cm^2^ area and the average of three successive measurements was taken for each sample. 

### 2.2. Characterization and Measurements

The morphology and dopant distribution were examined by atomic force microscope, namely with AFM (NT-MDT SOLVER NEXT, Moscow, Russia). Transmission spectra of the prepared films were measured at the normal incidence by using a double-beam spectrophotometer UV/Vis/NIR (JASCO, model V–770 ST, Portland, OR, USA) in the wavelength range of 300–1000 nm. The spectrophotometer was equipped with software for calculating chromaticity coordinates from the reflection spectra for the specification of color quality according to CIE 1931. The fluorescence peak wavelength was determined by steady-state fluorescence measurements in the wavelength range of 400–900 nm using the spectrofluorometer (SHIMADZU, RF-6000 PC, Kyoto, Japan). The fluorescence decay time was measured by the time-resolved fluorescence technique, which was measured out using the second harmonic-generation (SHG) Nd: YAG LASER MODEL LQ 129 and third harmonic-generation (THG) MODEL LG 103 (SOLAR LASER SYSTEM, Minsk, Belarus). The signal amplitude was measured as a function of time by a two-channel digital real-time 400 MHz-2GS/s oscilloscope (Tektronix model TDS 380, Southfield, MI, USA). FT-IR transmittance spectra were recorded in the wavenumber range of 4000–400 cm^−1^ using a spectrophotometer (CARY 500 UV-VIS-NIR, Pittsburgh, PA, USA). 

## 3. Results and Discussion

**Figure 2:**
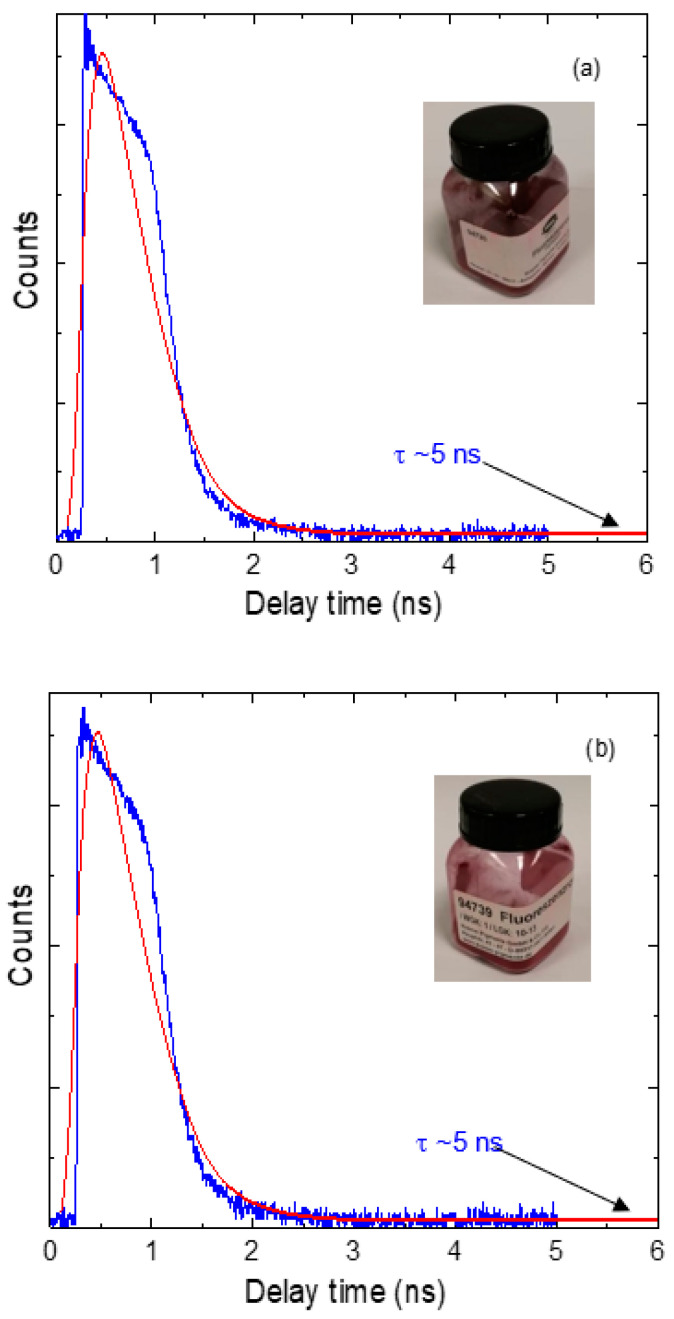
Time-resolved fluorescence decay curves for perylene dyes (**a**) KREMER 94720 and (**b**) KREMER 94739.

The fluorescence lifetime decay curves for the investigated perylene dyes are shown in Figure 2. It was observed that the value of the lifetime for the two dyes is five nanoseconds as determined directly from the curves. This result is in good agreement with our previous results about the excellent fluorescence efficiency of the investigated perylene dyes [5,26] because the fluorophore spends a short time in the excited state before returning to the ground state [39]. 

**Figure 3:**
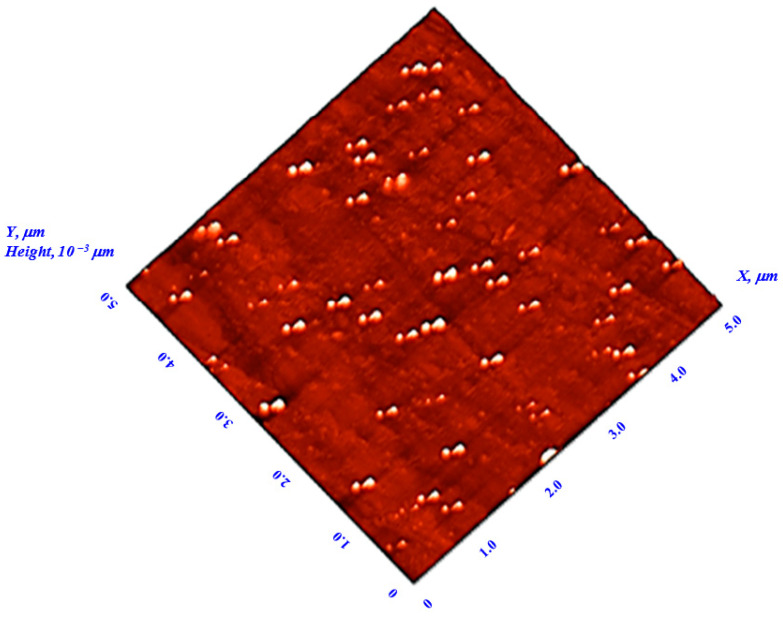
AFM micrograph of photoselective PMMA greenhouse film doped with 10^−1^ wt% perylene dye (KREMER 94720).

Figure 3 shows a representative AFM micrograph of photoselective PMMA greenhouse film doped with 10^−1^ wt% KREMER 94720; the image shows that the film surface is flat and homogenous. This proper morphology has been obtained for all the doping concentrations of perylene dyes as no burrs or pits had occurred in the surface structure. 

**Figure 4:**
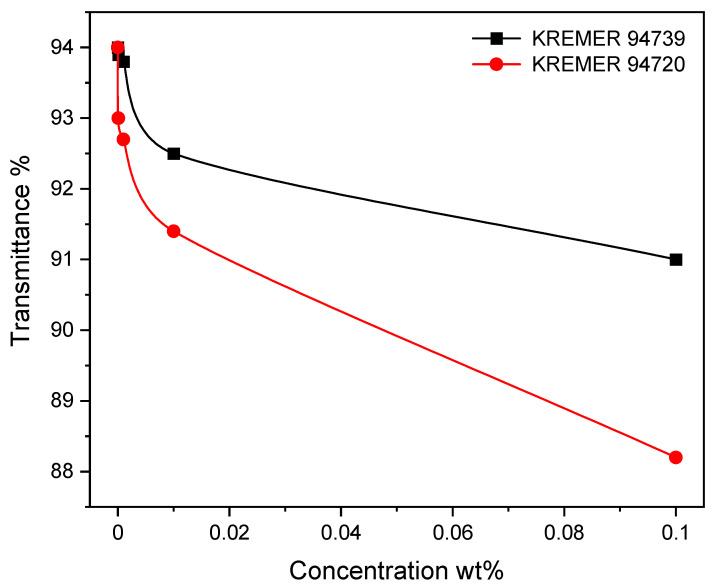
Effect of dye concentration on the optical transmittance for Photoselective PMMA greenhouse films doped with perylene dyes KREMER 94720 and KREMER 94739.

**Figure 5:**
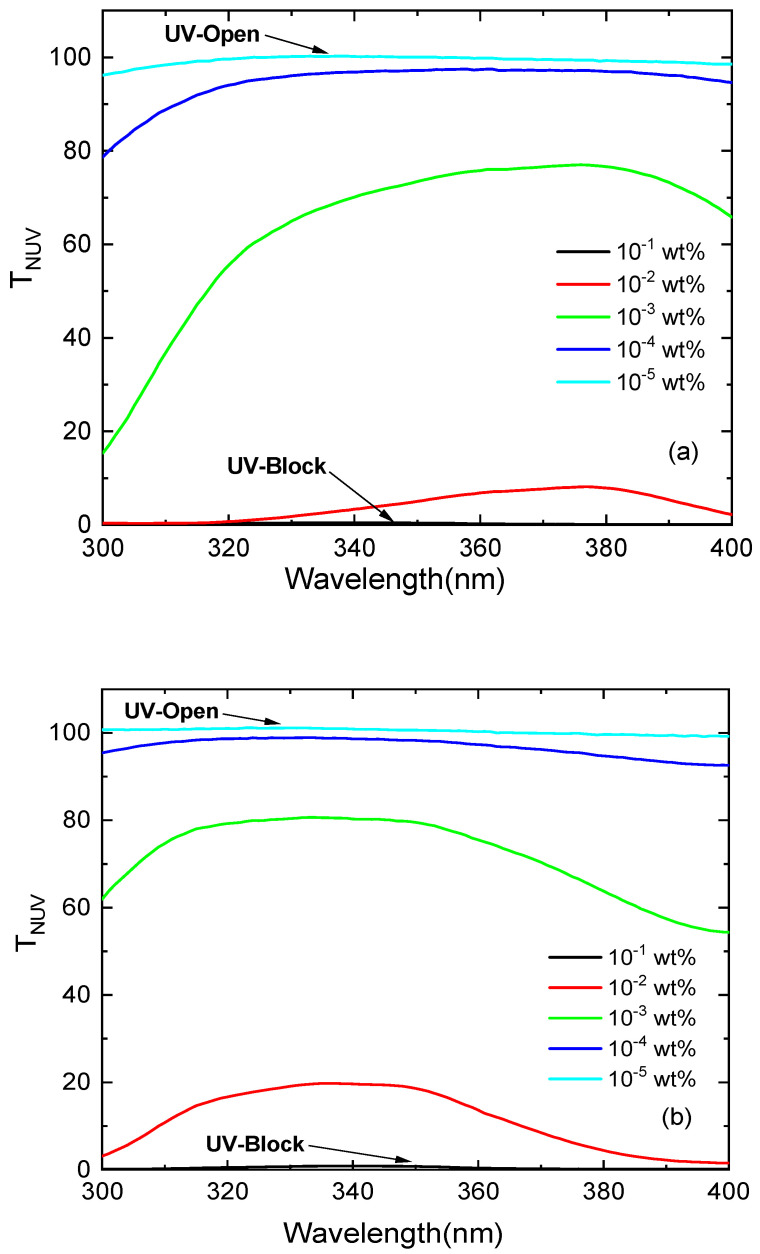
Transmission spectra in the NUV range for photoselective PMMA greenhouse films doped with perylene dyes (**a**) KREMER 94720 and (**b**) KREMER 94739.

The effect of the dye concentration on the optical transmittance for the investigated PMMA photoselective greenhouse films is shown in Figure 4. It is noted that all the films show high transmittance; this indicates that all the films have low opacity and thus raise the level of illumination required for the growing of the plants inside greenhouses. The values of the transmittance at the cut-off wavelength (600 nm) *T_cut-off_* were determined from the absorption spectra measured in reference and listed in Table 1. The transmission spectra in the near-UV range (300–400 nm), namely *T_NUV_*, is shown in Figure 5; two innovative, promising applications can be suggested for greenhouse photoselective films. The first application is the UV-block effect for the highest doping concentration 10^−1^ wt%, which blocks all the NUV radiation; this property is advantageous for reducing the blackening of the crops and the population of insects. The second application is the UV-open effect for the lowest doping concentration 10^−5^ wt% at which the films transmit more than 95% of the UV radiation. It is recognized that NUV radiation is not utilized for photosynthesis but very important to control photomorphogenesis. It is also necessary for the coloration of plants and some kinds of crops which require intense UV light to develop their characteristic color, especially red roses and fruits. 

**Figure 6:**
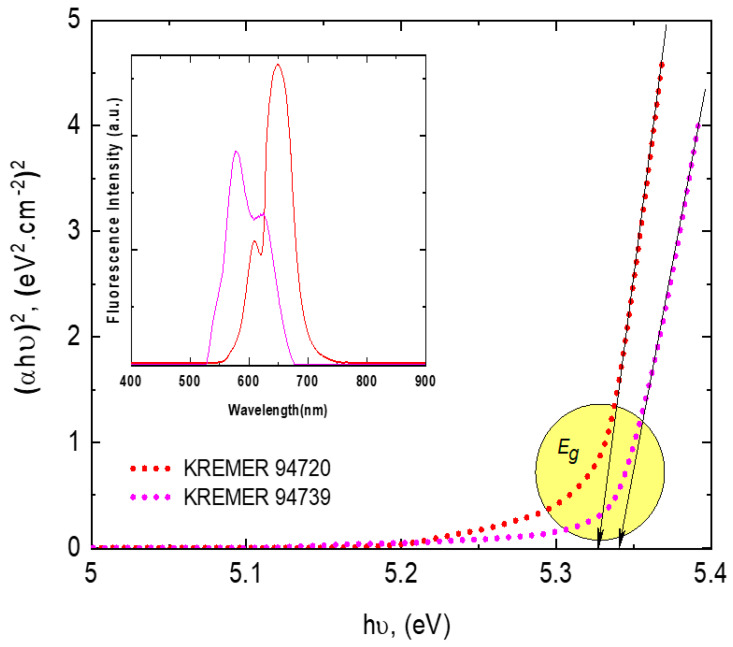
Correlation between the fluorescence spectra and inter-band transitions for photoselective PMMA greenhouse films doped with 0.1 wt% perylene dyes.

The bandgap, *E_g_*, of PMMA photoselective greenhouse films can be determined from the following equation using Tauc’s model [40,41,42,43].

(1) $$\alpha h\upsilon =A{\left(h\upsilon -E_{g}\right)}^{r}$$

where *α* is the absorption coefficient, *hυ* is the incident photon energy, *A* is a constant, and *E_g_* is the direct/indirect bandgap depending on the values of *r*, i.e., *r* = 2 for an indirect bandgap and *r* = 1/2 for a direct band. A representative plot of ${\left(\alpha E\right)}^{2}$ as a function of the photon energy (E) is shown in Figure 6, wherein the straight-line nature of the plots confirms that the optical transition in the films is directly allowed [44]. The values of the direct bandgap energy *E_g_* and the fluorescence peak wavelength *λ_f_* are listed in Table 2. The value of *E_g_* is slightly decreased by increasing the dye concentration; this behavior reflects the modification of the electronic structure of the PMMA host matrix. This considerable variation can be attributed to the appearance of different defect levels in the band structure of the host polymer matrix since the density of states is directly proportional to the concentration of the matrix defects [44,45]. This behavior is well correlated to the observed shift in the fluorescence peak values *λ_f_* as a result of the formation of layered H-aggregates, which led to the promising physical properties of the absorptive filter [41].

**Figure 7:**
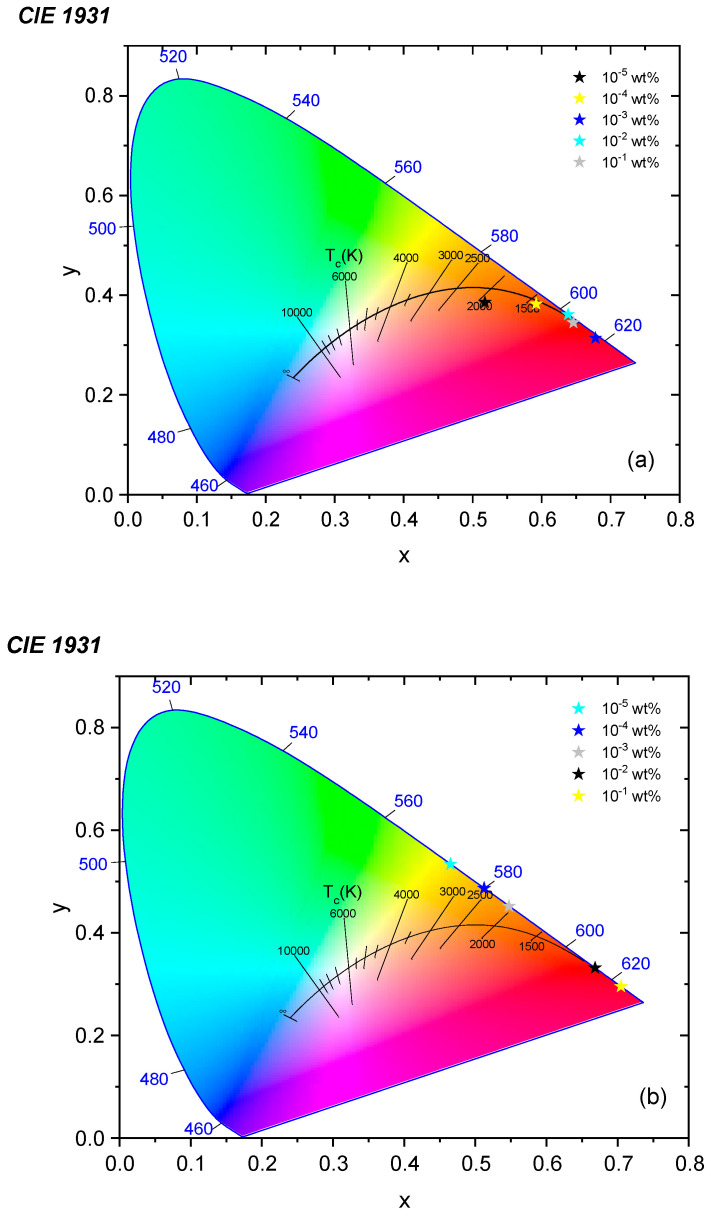
Chromaticity diagram of photoselective PMMA greenhouse films doped with (**a**) KREMER 94720 and (**b**) KREMER 94739.

The chromaticity coordinates have evaluated the color quality for the optimized UV-block and UV-open PMMA photoselective greenhouse films, as well as for CIE 1931, as plotted in Figure 7. This study is essential for the determination of color wavelength distributions in the visible electromagnetic spectrum and for the colors physiologically perceived by the human eye. It is observed that most of the photoselective films have pure spectral colors related to the fluorescence peak wavelength of each doping concentration, as measured in our previous work [26]. Besides, the intense red coloring has been obtained for the UV-blocking films doped with 10^−1^ wt%; this significant feature applies to the use of this film in photoselective greenhouse claddings in order to modify the sunlight spectrum entering the greenhouse and to control plant growth behaviors, specifically photosynthesis and photomorphogenesis. 

**Figure 8:**
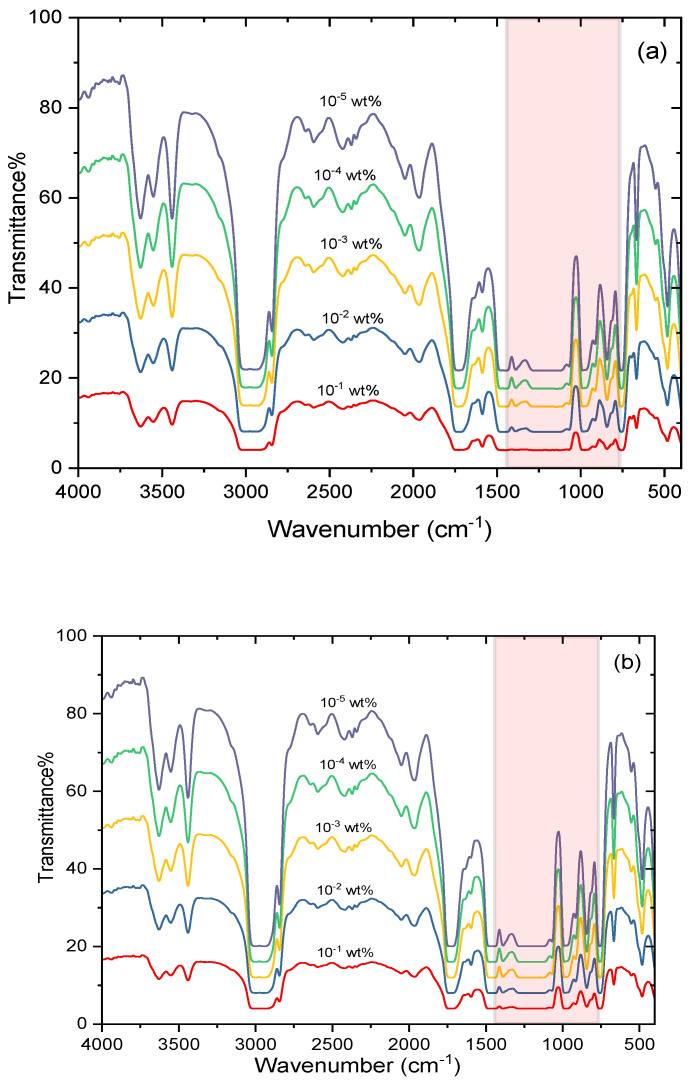
FT-IR transmission spectra for photoselective PMMA greenhouse films doped with perylene dyes (**a**) KREMER 94720 and (**b**) KREMER 94739; the shaded pink area represents the range 7–13 µm for calculating the infrared efficiency.

Figure 8 represents the FT-IR transmission spectra of PMMA photoselective greenhouse films in the wavenumber range of 1428–769 cm^−1^ corresponding to the infrared wavelength range of 7–13 µm; the infrared efficiency can be calculated in this range by

*η*_*IR* =_ [(*A*_100_ − *A_t_*)/*A*_100_] × 100 (2)

where *A*_100_ is the area under the transmission spectrum of a fully transmitting film in the infrared range of 7–13 µm and *A_t_* is the area under the IR spectrum of the tested film. The values of *η_IR_* are listed in Table 1. The values of all the films have good IR efficiency in the range of 72–78% as they absorb the infrared radiation and trap it to reduce the heat losses during the night, especially for winter crops. This result indicates that the highest doping concentrations have the best thermic effect by which the infrared radiation is absorbed and the heat losses are reduced during the night. These films have several advantages such as reduced energy consumption, smoother temperature drop, higher night temperature, better crop quality, and increased production during cold climates [6].

## 4. Conclusions

The present work represented extensive studies for evaluating novel spectroscopic properties of red-pigmented PMMA photoselective greenhouse films. The first property is the UV management as the best UV-open and UV-block effects were achieved by controlling the doping concentrations. The UV-open effect allows the near-ultraviolet radiation to enter the greenhouse in a self-controlled way; this effect is primarily directed to red roses and fruits, which require a specific range of ultraviolet radiation to grow. It is also crucial for the photomorphogenesis process and crop coloration due to its effect on the creation of the anthocyanin pigment. The advantage of the UV-block effect is the protection of crops from damage, especially in hot climate regions such as KSA, in addition to integrated pest management, which is an environmentally safe method to reduce the need for pesticides. The second property is the thermic effect which was obtained at the highest doping concentrations. The importance of these results is that the films absorb infrared radiation in the range and provide a reduction of the dramatic heat loss during the night, especially during winter and in cold countries.

## Figures and Tables

**Table 1:** Optical transmission spectra at the cut-off wavelength *T_cut-off_* (*%*) and infrared efficiency *η_IR_* (*%*) for photoselective PMMA greenhouse films doped with perylene dyes KREMER 94720 and KREMER 94739.

Concentration wt%	*T_cut-off_* (*%*)		*η_IR_* (*%*)	
	KREMER 94720	KREMER 94739	KREMER 94720	KREMER 94739
10^−5^	98.31	96.67	68.53	61.17
10^−4^	94.79	92.22	70.32	64.24
10^−3^	64.34	71.95	72.17	67.28
10^−2^	4.02	11.55	75.46	70.83
10^−1^	0.19	0.38	77.81	72.18

**Table 2:** Correlation between the optical bandgap *E_g_* and the fluorescence peak *λ_f_* for photoselective PMMA greenhouse films.

Concentration wt%	*E_g_* (eV)		*λ_f_* (nm)	
	KREMER 94720	KREMER 94739	KREMER 94720	KREMER 94739
10^−5^	5.32	5.34	583.84	569.81
10^−4^	5.29	5.32	604.32	574.30
10^−3^	5.12	5.30	649.17	577.92
10^−2^	5.22	5.27	607.68	628.84
10^−1^	5.27	5.24	613.10	644.41

## References

[1] Hussain I., Hamid H. (2003). Plastics in agriculture. Plastics and the Environment.

[2] Hammam M., El-Mansy M., El-Bashir S., El-Shaarawy M. (2007). Performance evaluation of thin-film solar concentrators for greenhouse applications. Desalination.

[3] Giacomelli G.A., Roberts W.J. (1993). Greenhouse covering systems. HortTechnology.

[4] El-Bashir S., Al-Harbi F., Elburaih H., Al-Faifi F., Yahia I. (2016). Red photoluminescent PMMA nanohybrid films for modifying the spectral distribution of solar radiation inside greenhouses. Renew. Energy.

[5] El-Bashir S., Al-Jaghwani A. (2020). Perylene-doped polycarbonate coatings for acrylic active greenhouse luminescent solar concentrator dryers. Results Phys..

[6] Waaijenberg D. Design, construction and maintenance of greenhouse structures. Proceedings of the International Symposium on Greenhouses, Environmental Controls and In-House Mechanization for Crop Production in the Tropics 710.

[7] Hiscott D., Cvetkovska M., Mumin M.A., Charpentier P.A. (2021). Light Downshifting ZnO-EVA Nanocomposite Greenhouse Films and Their Influence on Photosynthetic Green Algae Growth. ACS Appl. Polym. Mater..

[8] Sánchez-Lanuza M.B., Menéndez-Velázquez A., Peñas-Sanjuan A., Navas-Martos F.J., Lillo-Bravo I., Delgado-Sánchez J.M. (2021). Advanced Photonic Thin Films for Solar Irradiation Tuneability Oriented to Greenhouse Applications. Materials.

[9] Katsoulas N., Bari A., Papaioannou C. (2020). Plant responses to UV blocking greenhouse covering materials: A review. Agronomy.

[10] Hemming S., van Os E., Hemming J., Dieleman J. (2006). The effect of new developed fluorescent greenhouse films on the growth of Fragaria x ananassa ‘Elsanta’. Eur. J. Hortic. Sci..

[11] Pearson S., Wheldon A., Hadley P. (1995). Radiation transmission and fluorescence of nine greenhouse cladding materials. J. Agric. Eng. Res..

[12] Lamnatou C., Chemisana D. (2013). Solar radiation manipulations and their role in greenhouse claddings: Fluorescent solar concentrators, photoselective and other materials. Renew. Sustain. Energy Rev..

[13] Kumar M., Haillot D., Gibout S. (2022). Survey and evaluation of solar technologies for agricultural greenhouse application. Sol. Energy.

[14] Papakonstantinou I., Portnoi M., Debije M.G. (2021). The hidden potential of luminescent solar concentrators. Adv. Energy Mater..

[15] Meinardi F., Colombo A., Velizhanin K.A., Simonutti R., Lorenzon M., Beverina L., Viswanatha R., Klimov V.I., Brovelli S. (2014). Large-area luminescent solar concentrators based on ‘Stokes-shift-engineered’nanocrystals in a mass-polymerized PMMA matrix. Nat. Photonics.

[16] Weber W., Lambe J. (1976). Luminescent greenhouse collector for solar radiation. Appl. Opt..

[17] Swartz B., Cole T., Zewail A. (1977). Photon trapping and energy transfer in multiple-dye plastic matrices: An efficient solar-energy concentrator. Opt. Lett..

[18] Goetzberger A., Wittwer V. (1979). Fluorescent planar collector-concentrators for solar energy conversion. Festkörperprobleme 19.

[19] Batchelder J., Zewail A., Cole T. (1981). Luminescent solar concentrators. 2: Experimental and theoretical analysis of their possible efficiencies. Appl. Opt..

[20] Hermann A.M. (1982). Luminescent solar concentrators—A review. Sol. Energy.

[21] Friedman P., Parent C. (1987). Luminescent Solar Concentrator Development.

[22] Seybold G., Wagenblast G. (1989). New perylene and violanthrone dyestuffs for fluorescent collectors. Dye. Pigment..

[23] Ivri J., Burshtein Z., Miron E., Reisfeld R., Eyal M. (1990). The perylene derivative BASF-241 solution as a new tunable dye laser in the visible. IEEE J. Quantum Electron..

[24] Johansson L., Langhals H. (1991). Spectroscopic studies of fluorescent perylene dyes. Spectrochim. Acta Part A Mol. Spectrosc..

[25] Griffini G. (2019). Host matrix materials for luminescent solar concentrators: Recent achievements and forthcoming challenges. Front. Mater..

[26] El-Bashir S., AlSalhi M., Al-Faifi F., Alenazi W. (2019). Spectral properties of PMMA films doped by perylene dyestuffs for photoselective greenhouse cladding applications. Polymers.

[27] Raeisossadati M., Moheimani N.R., Parlevliet D. (2020). Red luminescent solar concentrators to enhance *Scenedesmus* sp. biomass productivity. Algal Res..

[28] Raeisossadati M., Moheimani N.R., Parlevliet D. (2019). Red and blue luminescent solar concentrators for increasing Arthrospira platensis biomass and phycocyanin productivity in outdoor raceway ponds. Bioresour. Technol..

[29] Cambié D., Dobbelaar J., Riente P., Vanderspikken J., Shen C., Seeberger P.H., Gilmore K., Debije M.G., Noël T. (2019). Energy—Efficient solar photochemistry with luminescent solar concentrator based photomicroreactors. Angew. Chem..

[30] Li Y., Sun Y., Zhang Y. (2019). Luminescent solar concentrators performing under different light conditions. Sol. Energy.

[31] Meinardi F., Bruni F., Brovelli S. (2017). Luminescent solar concentrators for building-integrated photovoltaics. Nat. Rev. Mater..

[32] Morandin L., Laverty T., Kevan P., Khosla S., Shipp L. (2001). Bumble bee (*Hymenoptera: Apidae*) activity and loss in commercial tomato greenhouses. Can. Entomol..

[33] Costa H.S., Robb K.L. (1999). Effects of ultraviolet-absorbing greenhouse plastic films on flight behavior of Bemisia argentifolii (*Homoptera: Aleyrodidae*) and Frankliniella occidentalis (*Thysanoptera: Thripidae*). J. Econ. Entomol..

[34] Díaz B.M., Biurrún R., Moreno A., Nebreda M., Fereres A. (2006). Impact of ultraviolet-blocking plastic films on insect vectors of virus diseases infesting crisp lettuce. HortScience.

[35] Lamnatou C., Chemisana D. (2013). Solar radiation manipulations and their role in greenhouse claddings: Fresnel lenses, NIR-and UV-blocking materials. Renew. Sustain. Energy Rev..

[36] Raviv M. (1988). The use of photoselective cladding materials as modifiers of morphogenesis of plants and pathogens. Int. Symp. Prot. Cultiv. Ornam. Mild Winter Clim..

[37] Espi E., Salmeron A., Fontecha A., García Y.A. (2006). Real, Plastic films for agricultural applications. J. Plast. Film. Sheeting.

[38] Garcia-Alonso Y., Espi E., Salmeron A., Fontecha A., Gonzalez A., Lopez J. (2006). New cool plastic films for greenhouse covering in tropical and subtropical areas. Int. Symp. Greenh. Cool..

[39] Lakowicz J.R. (2013). Principles of Fluorescence Spectroscopy.

[40] El-Bashir S., Alenazi W., AlSalhi M. (2017). Optical dispersion parameters and stability of poly (9, 9′-di-n-octylfluorenyl-2.7-diyl)/ZnO nanohybrid films: Towards organic photovoltaic applications. Mater. Res. Express.

[41] El-Bashir S., Yahia I., Binhussain M., AlSalhi M. (2017). Designing of PVA/Rose Bengal long-pass optical window applications. Results Phys..

[42] El-Bashir S., Yahia I., Binhussain M., AlSalhi M. (2017). Design of Rose Bengal/FTO optical thin film system as a novel nonlinear media for infrared blocking windows. Results Phys..

[43] Tauc J. (1970). Absorption edge and internal electric fields in amorphous semiconductors. Mater. Res. Bull..

[44] Tauc J. (1974). Optical properties of amorphous semiconductors. Amorphous and Liquid Semiconductors.

[45] El-Bashir S. (2012). Photophysical properties of fluorescent PMMA/SiO_2_ nanohybrids for solar energy applications. J. Lumin..

